# New Eco-Friendly Thermal Insulation and Sound Absorption Composite Materials Derived from Waste Black Tea Bags and Date Palm Tree Surface Fibers

**DOI:** 10.3390/polym16212989

**Published:** 2024-10-25

**Authors:** Mohamed Ali, Redhwan Almuzaiqer, Khaled Al-Salem, Hassan Alshehri, Abdullah Nuhait, Abdullah Alabdullatif, Abdulrahman Almubayrik

**Affiliations:** Mechanical Engineering Department, College of Engineering, King Saud University, P.O. Box 800, Riyadh 11421, Saudi Arabia; ralmuzaiqer@ksu.edu.sa (R.A.); kalsalem@ksu.edu.sa (K.A.-S.); hashehri@ksu.edu.sa (H.A.);

**Keywords:** natural thermal insulation materials, noise reduction coefficient, agro-waste utilization, three-point bending moment, thermogravimetric analysis, moisture content

## Abstract

A tremendous amount of waste black tea bags (BTBs) and date palm surface fibers (DPSFs), at the end of their life cycle, end up in landfills, leading to increased pollution and an increase in the negative impact on the environment. Therefore, this study aims to utilize these normally wasted materials efficiently by developing new composite materials for thermal insulation and sound absorption. Five insulation composite boards were developed, two were bound (BTB or DPSF with polyvinyl Acetate resin (PVA)) and three were hybrids (BTB, DPSF, and resin). In addition, the loose raw waste materials (BTB and DPSF) were tested separately with no binder. Thermal conductivity and sound absorption coefficients were determined for all boards. Thermal stability analysis was reported for the components of the tea bag (string, label, and bag) and one of the composite hybrid boards. Mechanical properties of the boards such as flexural strain, flexural stress, and flexural elastic modulus were determined for the bound and hybrid composites. The results showed that the thermal conductivity coefficients for all the hybrid composite sample boards are less than 0.07 at the ambient temperature of 24 °C and they were enhanced as the BTB ratio was reduced in the hybrid composite boards. The noise reduction coefficient for bound and all hybrid composite samples is greater than 0.37. The composite samples are thermally stable up to 291 °C. Most composite samples have a high flexure modulus between 4.3 MPa and 10.5 MPa. The tea bag raw materials and the composite samples have a low moisture content below 2.25%. These output results seem promising and encouraging using such developed sample boards as eco-friendly thermal insulation and sound absorption and competing with the synthetic ones developed from petrochemicals in building insulation. Moreover, returning these waste materials to circulation and producing new eco-friendly composites can reduce the number of landfills, the level of environmental pollution, and the use of synthetic materials made from fossil resources.

## 1. Introduction

Worldwide in 2020, seven million tons of tea were produced [1]. The annual growth rate of black tea production is expected to increase by 2.2% to reach 4.42 million tons in 2027 [2]. According to another source [3] the world consumes from 18 to 20 billion cups of tea daily. Negi et al. [4] have shown that 190,000 tons of tea waste were generated due to 857,000 tons of tea produced in India. Consumption of this huge amount of tea either loose or in the form of tea bags creates a tremendous amount of waste. This waste will end up in garbage bins, or landfill, or may be burnt. About 25% of those tea bags is polypropylene for bag sealing. Accordingly, this will pollute, damage, and create an environmental problem. Another common agricultural waste is date palm surface fibers. According to the Food and Agriculture Organization of the United Nations [5], Saudi Arabia produces 1.2 million tons of dates annually. Those date palm trees develop a tremendous amount of waste during their trimming time every year. One of those wastes is date palm surface fibers, which if not utilized efficiently will have a large environmental impact, especially if burnt, as usually happens in some parts of the world [6]. Saudi Arabia generates five hundred thousand tons of date palm waste annually [7]. Among these wastes, one hundred thousand tons are from leaves and fifteen thousand tons are from the date palm surface fibers [8,9,10]. In addition, both black tea bags (BTBs) and date palm surface fibers (DPSFs) could have good economic benefits if utilized efficiently since they are biodegradable, eco-friendly, natural, and sustainable. Therefore, the current study is focused on using both BTBs and DPSFs as new novel raw material polymers and their composites for thermal insulation and sound absorption.

Hussien et al. [11] have studied using tea waste as an additive with different percentages in unfired clay brick mixtures to improve their physical and mechanical properties. Ibrahim et al. [12] investigated new ceramic bricks made of zeolitic tuff with a small additive percentage of tea waste to develop their physical and mechanical characteristics as building materials. A similar study has been conducted by Ozturk et al. [13]. Anjum et al. [14] have shown that incorporating tea waste with a small percentage in a clay sample reduced its thermal conductivity from 0.54 to 0.3 W/(m K). Another way of using black tea waste is to extract the microcrystalline cellulose through microwave heating instead of using conventional heating and avoiding the traditional acid hydrolysis method as explained by Debnath et al. [15]. The tea bags were not tested for acoustic control. However, three different grades of spent tea leaves were studied in terms of sound absorption coefficient (SAC) by Wong et al. [16]. They found that the SAC of all sample leaves reached a maximum above 0.7 at a frequency range of 1993–3861 Hz. In a similar study by Ersoy and Kücük [17], SAC was observed to increase at a frequency range of 500–6300 Hz to a maximum of 0.6.

Ali et al. [18] have recently developed new composites made of DPSFs and pineapple leaf fibers (PALFs) using wood adhesive as a binder. Their results showed average thermal conductivity coefficients of 0.054–0.070 W/(m K) for the composite of DPSF, PALF, and the binder. These hybrid composites also had good sound absorption coefficients greater than 0.5 for frequencies greater than 1000 HZ. On the other hand, the bound composites of DPSF and the binder had a SAC > 0.5 for almost all frequencies greater than 300 Hz. DPSF was used with polystyrene to form new thermal insulation composite materials for buildings by Raza et al. [19]. The thermal conductivity coefficient of the composite having 20% DPSF was 0.053 W/(m K). In another study, Raza et al. [20] developed four samples for thermal insulation made of DPSF with different densities using polyvinyl alcohol as a binder. The average thermal conductivity coefficients of the samples were 0.038–0.051 W/(m K). New thermal insulation boards have been developed from DPSF with different densities using cornstarch as a binder by Ali and Abdelkareem [21]. The board’s average thermal conductivity coefficients were between 0.0475 and 0.0697 W/(m K). DPSF was hybridized with apple of Sodom fibers and different binders such as wood adhesive, cornstarch, and white cement to form new composites used for thermal insulation by Alabdulkarem et al. [22]. The developed boards had average thermal conductivity coefficients of 0.04234–0.05291 W/(m K). Their boards were also tested for sound absorption in the communication frequency range up to 2000 Hz. Their result showed that adding apple of Sodom fibers to the DPSF enhances the SAC over that of pure DPSF for any kind of binder. The SAC for the hybrid composite of DPSF and apple of Sodom fibers was found to be greater than 0.5 for a frequency range of 800–2000 Hz for all used samples.

On the other hand, Fouladi et al. [23] have measured the sound absorption coefficients for some natural fibers such as dry grass (SAC = 0.14 and 0.98 at 500 Hz and 2000 Hz, respectively), corn (SAC = 0.16 and 0.81 at 500 Hz and 2000 Hz, respectively), coir (SAC = 0.12 and 0.97 at 500 Hz and 2000 Hz, respectively), and sugar cane (SAC = 0.13 and 0.63 at 500 Hz and 2000 Hz, respectively) and found that these natural fiberboards could be used as alternatives for common building acoustic materials. In addition to that, Berardi and Iannace [24] have measured the noise reduction (NR) and sound absorption coefficients for some natural fibers such as sheep wool (NRC = 0.55 and 0.70 for thicknesses of 0.04 and 0.06 m, respectively), hemp (NRC = 0.4 for 0.03 m thickness), coconut (NRC = 0.5 and 0.75 for thicknesses of 0.05 and 0.1 m, respectively), kenaf (NRC = 0.55 and 0.70 for thicknesses of 0.04 and 0.06 m, respectively), cane (NRC = 0.40 and 0.50 for thicknesses of 0.04 and 0.08 m, respectively), cork (NRC = 0.30 for 0.03 m thickness), and cardboard (NRC = 0.50 for 0.10 m thickness). Their results showed that both coefficients depended on porosity, thickness, and the density of the natural fibers and were recommended for use in buildings.

As seen from the preceding literature survey, wasted tea bags have not been tested as insulation materials or for acoustic control, however, wasted DPSF was experimentally proven to be good for both thermal insulation and sound absorption as a polymer or composite with other materials. This finding motivates the current research of testing the novelty of using wasted tea bags for their thermal insulation characteristic and sound absorption as a polymer or as a hybrid with DPSF to form different composites with different densities. Therefore, laboratory experiments were designed to develop new bound and hybrid composite boards made of BTBs and DPSF with polyvinyl acetate as a resin. The thermal conductivity and sound absorption coefficients were measured for these boards with other mechanical and thermal stability analyses. The experimental results are promising for these materials to be used for thermal insulation applications in buildings since they are biodegradable and eco-friendly. Therefore, they could, in the future, be a candidate to replace fossil-fuel-based synthetic and petrochemical thermal insulation materials.

## 2. Materials and Methods

**Figure 1:**
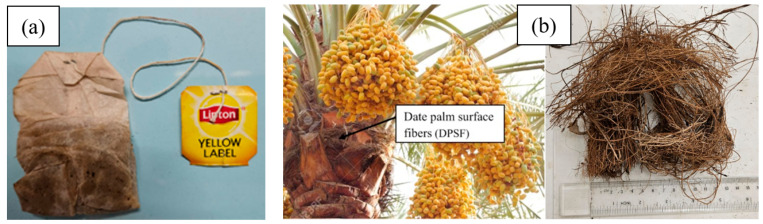
Raw wasted materials; (**a**) details of a tea bag showing the string, bag, label, and two staple pins, and (**b**) date palm surface fibers and their cut pieces.

Used black tea bags (BTBs) were collected from the nearby cafes and the households of the authors’ team. These tea bags were washed and dried using an electrical convection oven in the laboratory at 90 °C for 12 h, allowing all the water to evaporate. It should be mentioned that each BTB was kept with its label, string, and two staple pins, one to fix the string to the label and the other to the bag as shown in Figure 1a. The date palm surface fibers (DPSFs) were collected from the agricultural authority during the trimming time at a specific time of the year. During this time, huge amounts of date palm surface fibers are normally discarded. Another source of the DPSFs was the nearby farms during their trimming time. The collected DPSFs were washed and dried in the same way as the tea bags. The DPSFs were cut to approximately 10–15 cm long. Figure 1b defines the DPSF and their cut pieces.

### 2.1. Loose Sample Polymer Preparation for Testing

**Figure 2:**
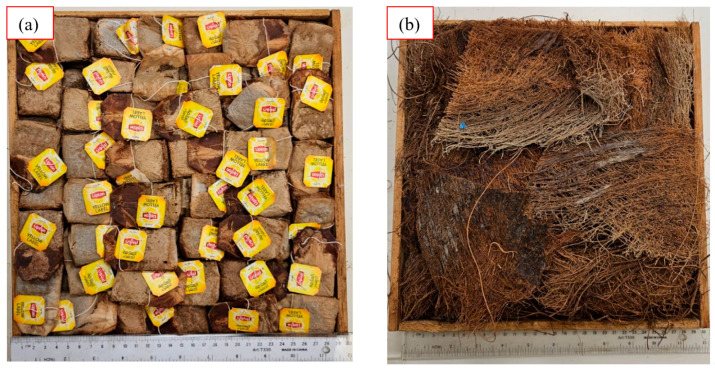
Loose raw materials in the wooden boxes, (**a**) BTBP # 1 and (**b**) DPSFP # 3.

The loose wasted BTBs and the DPSFs were enclosed in two wooden frame boxes with inside sizes of 28.5 × 28.5 × 4.1 cm^3^ as shown in Figure 2a,b, one for the BTBs and the other for the DPSFs. This frame size was chosen to be ready for fitting inside the heat flow meter for thermal conductivity coefficient measurements as will be explained in Section 4.

### 2.2. Composite Bound Sample Preparation for Testing

**Figure 3:**
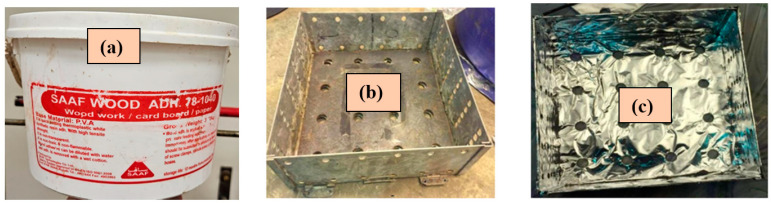
Resin and the stainless steel mold; (**a**) bucket of polyvinyl acetate (PVA) resin (wood adhesive), (**b**) the mold before covering with aluminum foil sheets, (**c**) after covering.

**Figure 4:**
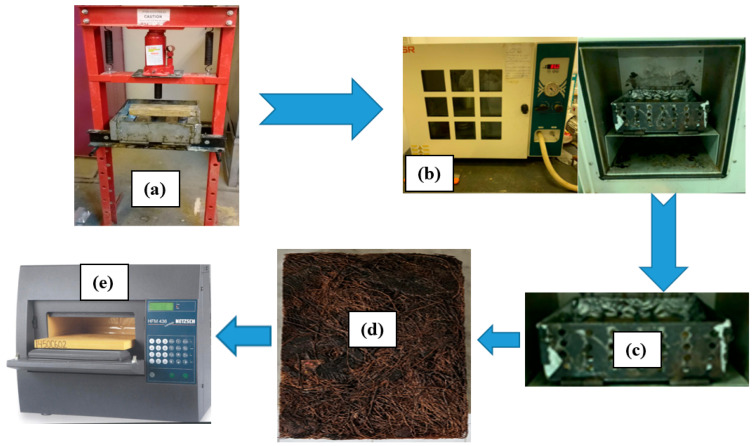
Sample preparation; (**a**) Pressing the sample; (**b**) convection oven; (**c**) cooling the mold with the board; (**d**) sample board; and (**e**) heat flow meter for thermal conductivity measurement.

Safe and non-toxic polyvinyl acetate (PVA) resin (wood adhesive, Figure 3a) was used to bind the loose raw materials of BTBs and DPSFs shown in Figure 2a,b. The ingredients of the resin with its chemical and physical properties can be obtained from [25]. A solution of resin and water was made on a mass basis with a ratio of one part of the resin to two parts of water. The loose raw materials were completely immersed in the resin solution to be sure that each BTB or DPSF was in contact with the resin solution. Two stainless steel molds of size 30 × 30 × H cm^3^ (Figure 3b,c) were used to make wetted samples, one for BTBs and the other for the DPSFs, where H is the height of each sample in the mold based on its thickness. The mold surfaces were covered with aluminum foil sheets to prevent the sample from sticking to the stainless steel mold. The mold was then moved to a hydraulic press to adjust the sample to a specified thickness from 20 to 50 mm according to each sample. After that, the stainless steel mold with the sample was moved to the convection oven for drying. The last step was to move the hot dried mold with the sample, keep it until it cooled down to the laboratory ambient temperature, remove the sample from the mold, and move it to a heat flow meter for thermal conductivity coefficient measurements. Figure 4 summarizes the sample preparation processes. Table 1 shows the specifications of the loose polymers and bound composites.

### 2.3. Composite Hybrid Sample Preparation for Testing

**Figure 5:**
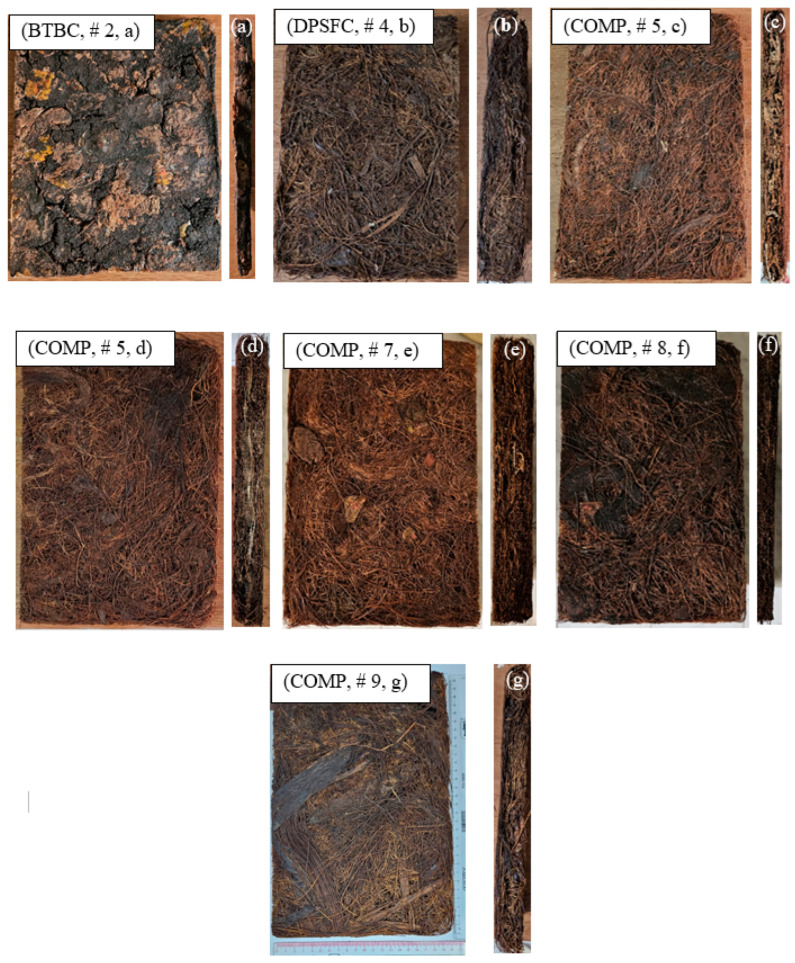
Plan and side view of the prepared samples; (**a**) bound composite of BTBs and resin, (**b**) bound composite of DPSFs and resin, and (**c**–**g**) hybrid composite of BTBs, DPSFs, and resin, see Table 1 for more details.

Hybrid composite means mixing BTBs with DPSFs with different compositions with the same binder used in Section 2.2 (PVA) using the same mold specified and shown in Figure 3. Table 1 shows the complete specifications, dimensions, percentage of each polymer in the sample, mass, volume, figure number of the sample, and the density of the samples. Figure 5 shows the real pictures of all prepared samples with the resin, either bound or hybrid composites.

## 3. Characterization

### 3.1. Mechanical Test for Bound and Hybrid Composite Samples

**Figure 6:**
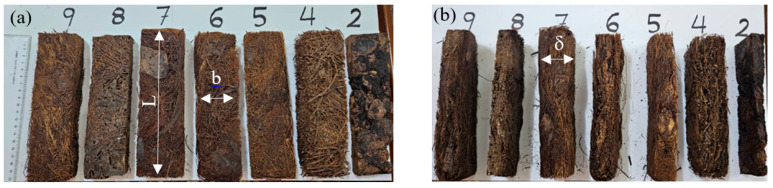
Cut specimens for the bending test; (**a**) Plan view and (**b**) side view.

**Figure 7:**
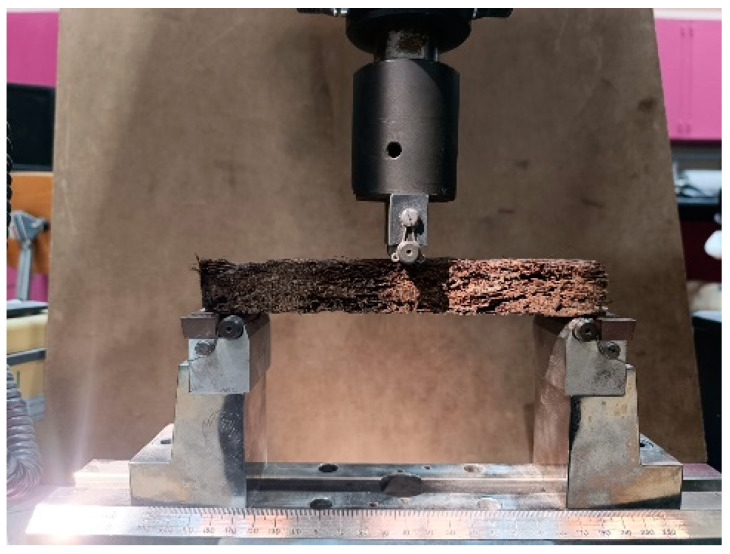
The universal testing machine (UTM, INSTRON 5984) used for the bending test.

The bending moment test (three-point) was obtained following the standard ASTM D790-03 [26] for all composite samples # 2, 4, 5, 6, 7, 8, and 9, either bound or hybrid, as shown in Table 1. The cut specimens for the test (Figure 6) have dimensions 20 × 5.0 × δ cm^3^, where δ is the thickness as defined in Table 2. The universal testing machine (UTM, INSTRON 5984) (Instron, Norwood, MA, USA) (Figure 7) in our mechanical engineering laboratory with a crosshead speed of 2 mm/min was used to determine the deflection D, the applied force *F*, flexural strain ϵ*_f_*, and flexural stress *σ_f_* as defined by Equation (1), where *E_f_* is the flexural elastic modulus. Table 2 specifies the other used dimensions.

(1) $$\sigma_{f}=\frac{3FL}{2b\;\delta^{\;2}},\;\epsilon_{f}=\frac{6\;D\delta }{L^{2}},\;E_{f}=\frac{L^{3}S}{4b{\delta \;}^{3}}$$

### 3.2. Thermal Conductivity Coefficient Measurement

A heat flow meter (Lambda, HFM 436 (NETZSCH-Gerätebau GmbH, Wittelsbacherstraße 42, 95100 Selb, Germany, Figure 4e) was used to determine the thermal conductivity coefficient for all samples: loose polymers (Figure 2a,b) and bound or hybrid composites (Figure 5a–g). The HFM follows the ASTM-C518 [27] standard testing method. The allowable size of the sample to be used by the HFM is 30 × 30 × δ cm^3^, where δ is a variable thickness up to 10 cm. A self-automated sensor provided by the HFM measures the thickness. The thermal conductivity coefficient and the temperature were measured up to an accuracy of ±1% to 3% W/(m K) and ±0.01 °C, respectively, as provided by the manufacturer of the HFM. The thermal conductivity coefficients were measured for all samples from 20 °C to 80 °C.

### 3.3. Sound Absorption Coefficient Measurement

**Figure 8:**
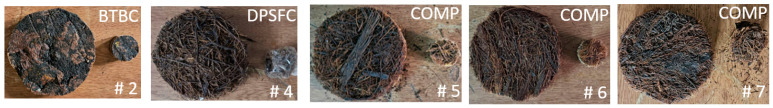
Samples for sound absorption coefficient measurement.

Two impedance tubes with inside diameters of 10 cm and 3 cm of BSWA (BSWA Technology Co. Ltd., Bejing, China) were used to measure the sound absorption coefficient (SAC). The large tube (10 cm) and the small one (3 cm) were used for a frequency range of 63–1600 Hz and 800–6300 Hz, respectively. Therefore, samples were prepared for this test with 10 cm and 3 cm diameters for bound and hybrid composites as shown in Figure 8 with specification details in Table 1. The impedance tubes’ principle of working and the position of microphones at each frequency range can be obtained from our previous publication, Ali et al. [28]. The BSWA software VA-Lab IMP, Version: V1.03, conforms to ISO 10534-1 [29] and ISO 10534-2 [30] standards.

### 3.4. Microstructure Analyses of the Black Tea Bags

The black tea bags were characterized by applying scanning electron microscopy (SEM) and energy dispersive X-ray (EDX) spectroscopy analysis.

#### 3.4.1. Scanning Electron Microscopy Analysis

Scanning electron microscopy (SEM) (JEOL; JSM7600F, Peabody, MA 01960, USA) was used to determine the surface morphology of the black tea bag sample (BTBP, # 1) at different magnifications. The tea bag sample must be oven-dried first and then coated with platinum to avoid any electrostatic charging, which may happen during the test.

#### 3.4.2. Energy Dispersive X-Ray (EDX) Spectroscopy Analysis

The chemical composition of the black tea bag was obtained using EDX analysis at different spots. This test gives qualitative results about the composition of the tea bag. It should be mentioned that the field-emission SEM (FE-SEM) (JEOL; JSM7600F) is equipped with EDX.

### 3.5. Thermal Stability of the Tea Bags

Thermal stability analysis was performed for the tea bag components such as string, bag, and label using thermogravimetric analysis (TGA) and its differential (DTGA). The test uses the SDT Q600 V20.9 Build 20 setup (New Castle, DE, USA), a TA instrument fitted with a nitrogen purge gas. The test was also performed for the composite sample # 7. The initially used mass for the tea bag, string line, label, and composite sample # 7 are 19.45 mg, 24.96 mg, 25.42 mg, and 7.96 mg, respectively. An alumina pan was used to heat the sample mass from room temperature up to 550 °C with a heating rate of 10 °C/min. The flow rate of the nitrogen gas was 100 mL/min. It should be noted that the thermal stability of the DPSF was described in our previous paper [21].

### 3.6. Moisture Content Test of the Tea Bags

**Figure 9:**
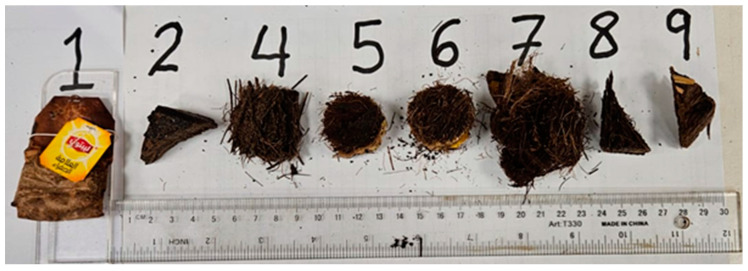
Samples used for moisture content determination; numbers refer to the sample number in Table 1.

Figure 9 presents the samples used for moisture content determination. These samples were first oven-dried for 8 h at 100 °C and, at the end of the drying time, the masses were recorded as *m*2. Then, the samples were left in the laboratory environment of relative humidity and temperature of 51.7% and 21.6 °C, respectively. The masses were measured every 5 min as *m*1 until the readings were steady. The amount absorbed by each sample was calculated as a percentage amount of that just after drying, *m*2, following Equation (2) of the standard ASTM D2974-07A [31].

(2) $$\%\;\mathrm{of}\;\mathrm{moisture}\;\mathrm{content}=\frac{m1-m2}{m2}\times 100$$

## 4. Results and Discussion

### 4.1. Three-Point Bending Moment

**Figure 10:**
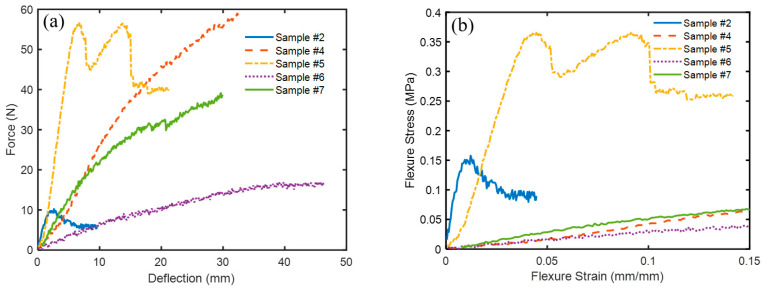
Profiles for the three-point bending test for bound and hybrid composites; (**a**) force–deflection and (**b**) stress–strain.

**Figure 11:**
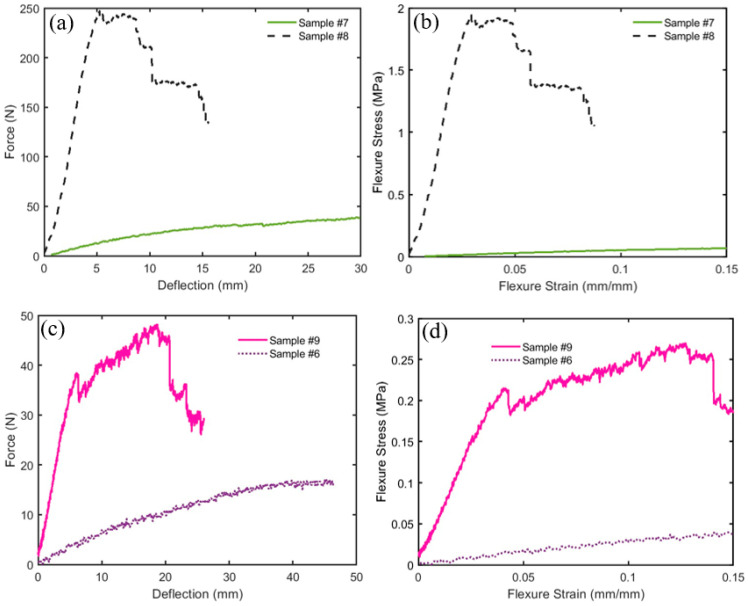
Profiles for the three-point bending test show the effect of increasing the density and the resin in the specimens on force–deflection and flexure stress–strain curves; (**a**,**b**) comparison between samples # 7 and # 8 and (**c**,**d**) comparison between samples # 9 and # 6.

**Figure 12:**
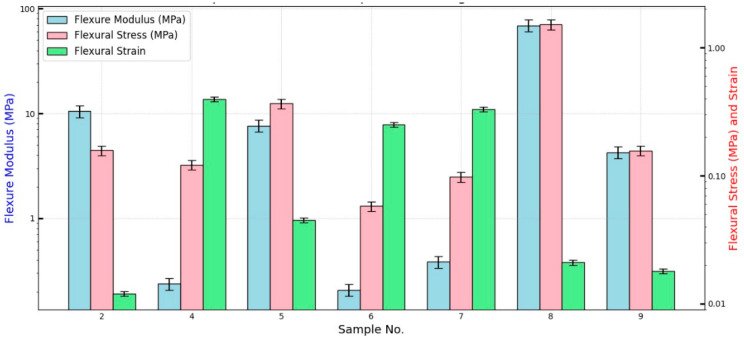
Bar chart presenting Flexure Modulus, Flexural Stress, and Flexural Strain for all samples.

Figure 10a,b show the force versus deflection and the flexure stress versus flexure strain, respectively, for the bound and hybrid composite samples # 2, 4, 5, 6, and 7. Table 3 shows flexure modulus, *E_f_*, flexural stress *σ_f_*, and flexural strain *ε_f_* as defined by Equation (1) for the composite samples. It should be noted that *σ_f_* presents its maximum at the end of the linear region following [32] as shown in Figure 10b. Furthermore, the flexural strain *ε_f_* is calculated at the maximum value of *σ_f_*. The slope S was calculated from the load–deflection curve in the linear zone (Figure 10a). By inspection of specimens # 2, 5, and 7 in Table 3, it is observed that the flexure modulus, *E_f_*, increases as the density increases from bound composite to hybrid composite, which agrees with the results of [33,34]. The degree of coherence (percent of the polymerized (PVA) resin in the specimen) presents an important factor in the flexure modulus *E_f_* and flexural stress *σ_f_*. Accordingly, as this percentage increases one would expect an enhancement of both *E_f_* and *σ_f_* as shown in Figure 11a–d when comparing samples # 7 and # 8 as a group and # 9 and # 6 as another group, respectively. Each group almost has an equal mass of polymers but with increased resin mass. Therefore, Figure 11a–d show that the density and the percentage of polymerized resin have an essential enhancement role in both *E_f_* and *σ_f_*. In addition, inspection of Table 3 and Figure 10 and Figure 11 indicates that composite specimen # 2 is the best among the bound ones and # 8, 5, and 9 present the best hybrid composites in descending order. Another factor affecting the flexure modulus is the material’s compactness (small thickness). Therefore, it could be concluded that as the density and the percentage of resin increase, a small thickness enhancement would be expected in the flexure modulus. Mechanical parameters’ comparisons are presented in logarithmic scale bar charts in Figure 12 for all samples.

### 4.2. Thermal Conductivity Measurements

**Figure 13:**
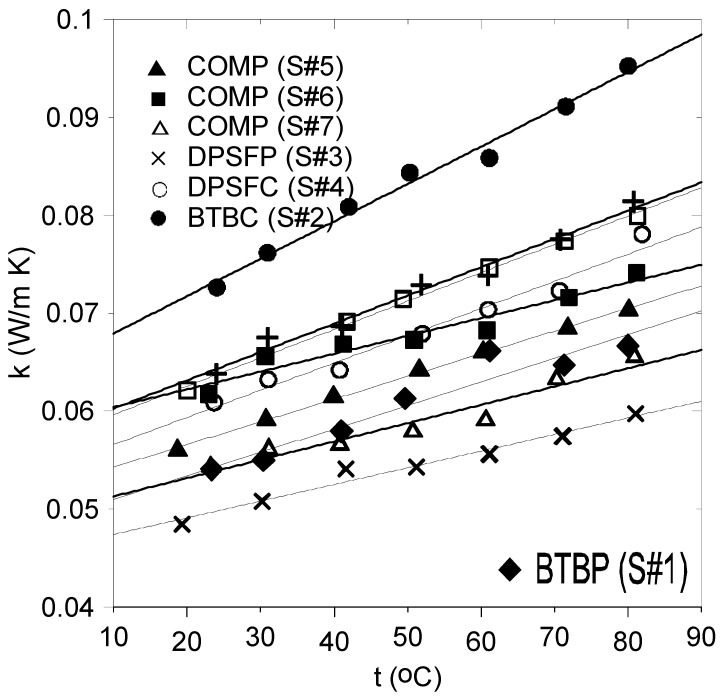
Thermal conductivity coefficient curves for samples # 1–7. Solid lines represent the fitting curves.

Figure 13 shows the thermal conductivity coefficient profiles for the samples # 1, 2, 3, 4, 5, 6, and 7. The solid lines through the data represent the linear fitting curves with details given in Table 4.

**Figure 14:**
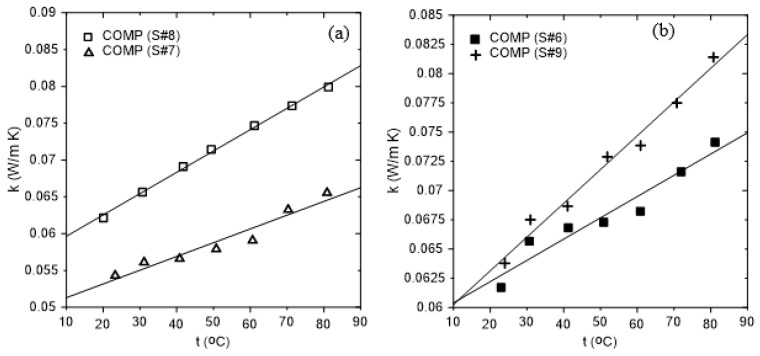
The effect of resin on thermal conductivity coefficient; (**a**) comparison between samples # 8 and # 7 and (**b**) between samples # 6 and # 9.

It should be noted that all values of the thermal conductivity coefficients at the ambient temperature of about 24 °C are less than 0.07 W/(m K), strongly suggesting that the samples can be used as thermal insulation materials. Furthermore, adding resin to the polymer and composite samples increases their thermal conductivity coefficient compared to pure polymer samples. This is clear by comparing the values of k of BTBP (♦) and its composite BTBC (●) and between DPSFP (**×**) and DPSFC (ο). Furthermore, adding DPSFP to form composite samples such as # 5, 6, or 7 enhanced their thermal conductivity coefficients compared to the bound BTBC sample # 2, keeping in mind the percentage of resin. Based on that, composite sample # 7 represents the best among all composite samples. Figure 14a,b were constructed to clarify the effect of increasing the percentage of resin in the sample. Figure 14a shows that effect, since samples # 7 and # 8 almost have the same polymer mass percentage but the main difference is in the percentage of resin. Sample # 7 has 24% but sample # 8 has 46%. The same applies to samples # 6 and # 9, where sample # 6 has 22% and sample # 9 has 32% resin as shown in Figure 14b.

Table 5 compares the thermal conductivity coefficient of this study’s developed boards to that of conventional synthetic insulation materials. Even though the thermal conductivity values of the synthetic materials are lower than those of the study’s developed boards, these boards are inexpensive, environmentally friendly, and economical compared to the synthetic ones. In addition, these boards have no CO_2_ emission, compared to that produced through the manufacturing process of the synthetic insulation materials due to the use of fossil fuels. It should be noted that Asdrubali et al. [35] have classified thermal insulation materials based on their thermal conductivity coefficient as good if it is lower than 0.0566 W/(m K), intermediate for 0.055 < k < 0.091 W/(m K), and poor for k > 0.091.

### 4.3. Sound Absorption and Noise Reduction Coefficients

**Figure 15:**
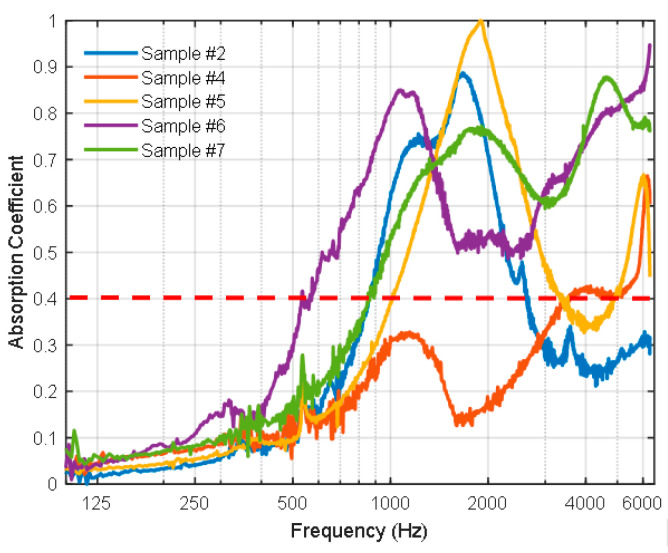
The sound absorption coefficient profiles for samples # 2, 4, 5, 6, and 7 at a frequency range 100–6000 Hz. Dashed line presents a borderline SAC = 0.4.

Figure 15 shows the sound absorption coefficients (SACs) for a wide range of frequencies (100–6000 Hz) for bound and hybrid composite samples # 2, 4, 5, 6, and 7. In the range of communication frequency up to 2000 Hz, it is observed that the bound composite sample # 2 of black tea bags has a SAC > 0.5 in the frequency range of 900–2000 Hz with a peak of SAC ≈ 0.9 at about 1800 Hz. Furthermore, the hybrid composite samples # 5, 6, and 7 have similarly good SAC. For example, # 5 has SAC > 0.4 for a range of 1000–2000 Hz with a peak of SAC ≈ 1 at 2000 Hz, # 6 has SAC > 0.5 for a range of 600–2000 Hz with a peak of SAC ≈ 0.85 at about 1200 Hz, and # 7 has SAC > 0.5 at a range of 900–2000 Hz with a peak SAC ≈ 0.76 at 1800 Hz. However, the bound composite sample # 4 has a good SAC > 0.4 for a frequency > 3500 Hz and it looks like it does not have a good SAC in the communication range. This could be attributed to the high airflow resistance, which indicates that the pores are close to each other and tend to obstruct the airflow, hence low SAC is expected [24]. It should be noted that the thickness, density, porosity, and percentage of polymerized binders in the board influence the value of SAC. Therefore, the presence of air passages in the boards helps absorb the sound waves and hence increase the SAC, however, if the boards have continuous pores or passages through their thickness, it allows for sound waves to pass and reduce the SAC. The percentage of polymerized binder in the sample controls its structural rigidity and hence the SAC values. The dashed horizontal line in Figure 15 presents a borderline SAC = 0.4 and, when the SAC exceeds this value, the boards are considered good sound absorbers [37]. The average values for a one-third octave of the sound absorption coefficients at frequencies 250, 500, 1000, and 2000 Hz were used to estimate the noise reduction coefficient (NRC). These values were rounded to the nearest 0.05 following [24,38]. Table 6 shows the values of the noise reduction coefficient (NRC) for the samples # 2, 4, 5, 6, and 7. Accordingly, as the NRC exceeds 0.2 or 0.4, the material is considered sound absorbing or has a practical value, respectively [39,40,41]. Therefore, samples # 2 and # 5 are considered good sound absorbers, and samples # 6 and # 7 are better yet and have practical value.

**Figure 16:**
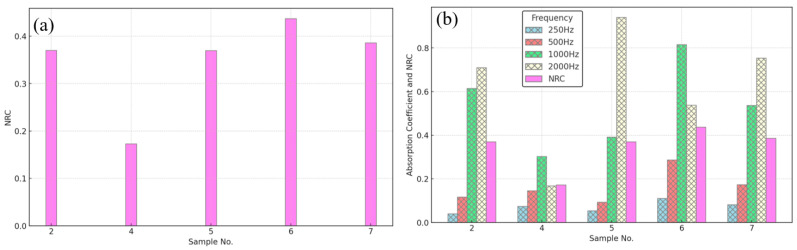
Noise reduction and sound absorption coefficient profiles for samples # 2, 4, 5, 6, and 7; (**a**) NRCs and (**b**) comparison between NRC and SAC at one-third octave values.

Figure 16a shows a bar chart of the noise reduction coefficient for bound and hybrid composite samples # 2, 4, 5, 6, and 7. Figure 16b compares the NRC of the samples and the SAC at one-third octave values. Inspection of SACs and the NRCs indicates that the above-developed bound composite sample of BTB (# 2) and its hybrid composites with DPSF, samples # 5, 6, and 7, have a better acoustic characteristic in absorbing the sound, which promotes them as suitable for building applications.

Table 7 compares the developed samples’ sound absorption and noise reduction coefficient with similar agricultural and synthetic insulation materials using the same technique, which are in the same order of magnitude as similar materials.

### 4.4. Microstructure of the Black Tea Bags

**Figure 17:**
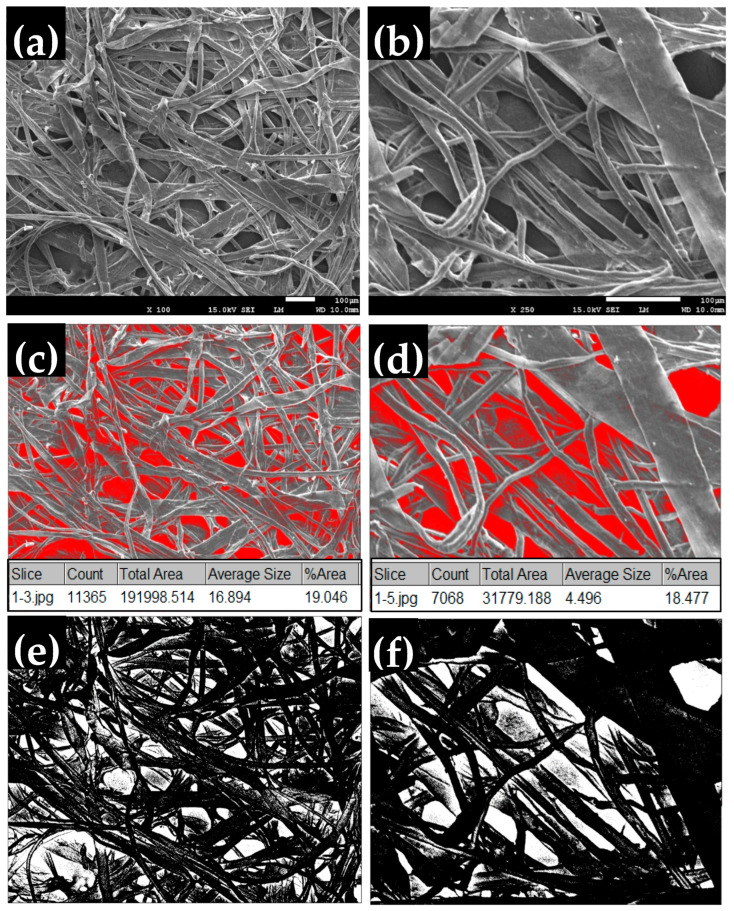
(**a**,**b**) Black tea bag texture shows the fibers and pores at two magnifications; 100× and 250×, (**c**–**f**) the porosity analysis using ImageJ software at the corresponding magnifications.

Figure 17a,b show the morphology analysis of a tea bag at two different magnifications (100 and 250) using scanning electron microscopy (SEM). The surface structure shows the external morphology of the tea bag’s texture. The texture has many pores as shown in the SEM images, marked as red spots in Figure 17c,d and white spots in Figure 17e,f. The BTB total surface optical porosity percentage was determined by using ImageJ software, version 1.8.0. Digital SEM photomicrographs (Figure 17a,b) were preprocessed and analyzed using this software. The analysis was performed two times using different thresholds, and then the average was calculated. The porosity of a given sample can be described as the percentage ratio of available pore area to the total analyzed area. The 2D porosity range was between 18 and 20% for the surface as shown in Figure 17c,d. Accordingly, the relative density of the BTB was calculated using the following relationship:

Relative Density = 1 − Porosity(3)

where porosity is expressed as a decimal (e.g., 18% porosity is 0.18). Based on this calculation, the relative density of the BTB is between 0.80 and 0.82.

**Figure 18:**
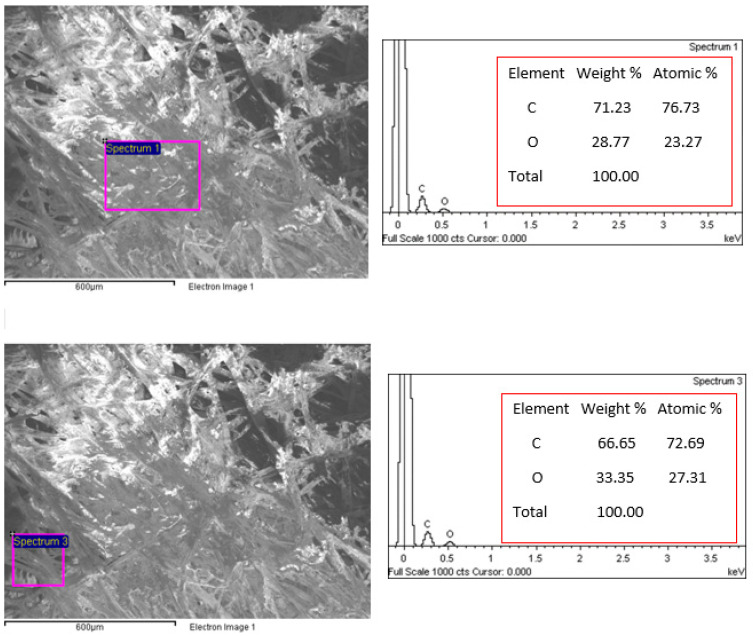
EDX analyses of the tea bag texture at two different spots.

Figure 18 shows the EDX analysis of the tea bag texture at two different spots. The constituents of the tea bag are carbon (66.65% to 71.23%) and oxygen (28.77% to 33.35%) and their percentages are different from one spot to another as shown in the figure.

### 4.5. Thermal Stability of the Tea Bags and Their Composites

**Figure 19:**
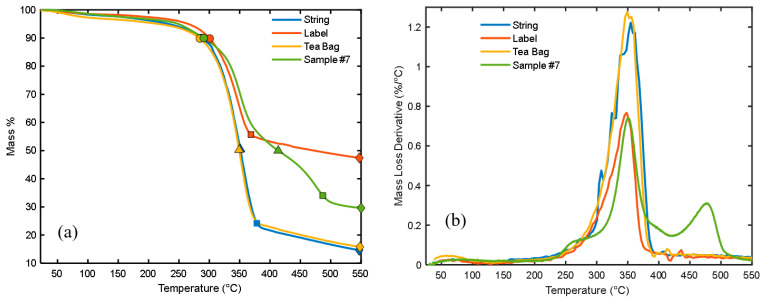
Thermal stability analysis for the tea bag, label, string, and composite sample # 7; (**a**) Thermogravimetric analysis (TGA) and (**b**) Differential thermogravimetric analysis (DTGA). Blue, orange, yellow, and green symbols present string, label, tea bag, and sample 7, respectively.

Figure 19a,b show the thermal stability analysis of the components of the tea bag such as the tea bag itself, label, and string. The decomposition of the composite sample # 7 is also shown in the figure for comparison. Thermogravimetric analysis (TGA) and its differential (DTGA) are presented in Figure 19a,b, respectively. Figure 19a indicates that all the tested components, tea bag, label, and string, and composite material of # 7 are thermally stable up to 284.5, 300.7, 287, and 291 °C, respectively, since they lost only about 10% of their mass, which is the water vapor in the components and the sample. These stable temperatures are comparable to those obtained by Alemdar and Sain [45] for untreated wheat straw fiber. The major points of decomposition and degradation of these components and sample # 7 are shown in Table 8. The major degradation temperature for almost all components and the composite sample ranges between 300 and 400 °C as demonstrated in Figure 19b. It should be noted that composite sample # 7 reached a char formation at 800 °C with a 20% mass. These degradation results agreed with the review study by Asim et al. [46] for most natural fibers.

### 4.6. Moisture Content of the Tea Bags

**Figure 20:**
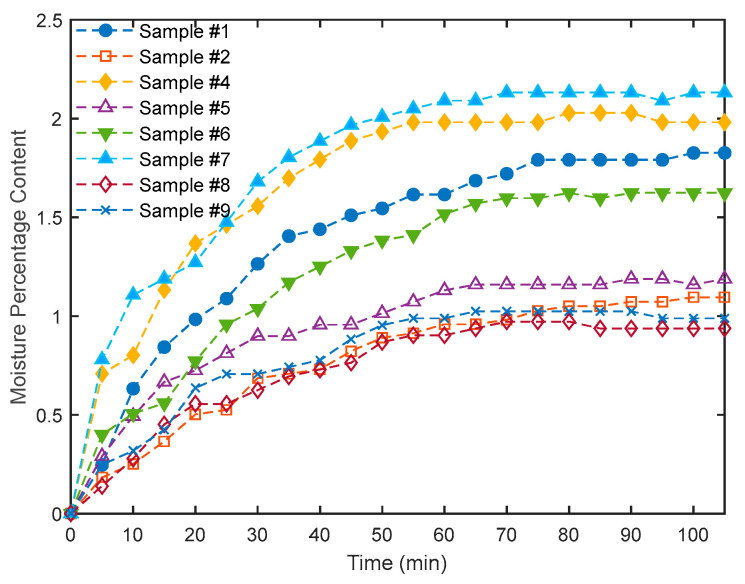
Moisture profiles for loose polymer BTB (sample # 1), bound composite samples (# 2 and # 4), and hybrid composite ones (# 5, 6, 7, 8, and 9).

Figure 20 shows the steady state profiles of the moisture content for the loose polymer BTB (# 1), bound BTB composite (# 4), bound DPSF composite, and the hybrid composites of both BTB and DPSF (# 5, 6, and 7) following Equation (2). Inspection of the figure indicates that the loose sample (BTB, # 1) absorbed more moisture, about 2.1%, than the bound sample (BTBC, # 2), which absorbed about 1.1%. This could be attributed to the fact that adding resin to the polymer blocked most of the pore spaces and hence reduced the composite’s ability to absorb the water. It is also observed that as the percentage of the DPSF increases in the composite samples (# 5, 6, and 7) the moisture content increases due to the increasing number of pores in the composites. Furthermore, as the percentage of resin increases in the composite, this leads to a decrease in the moisture content as shown for similar samples # 7 and # 8 and between samples # 6 and # 9. Accordingly, for all tested polymers and composites, the maximum percentage of moisture content is about 2.1%, which is much lower than the safe moisture content of 16% of similar natural straw fibers as recommended by Bainbridge [47].

## 5. Conclusions

New eco-friendly composite boards were developed on the laboratory scale from waste black tea bags (BTBs) and discarded date palm tree surface fibers (DPSFs) using polyvinyl acetate resin. These composites were tested to be used as thermal insulation and sound absorption materials to compete with synthetic ones developed from petrochemicals. The thermal conductivity coefficient of the composite bound BTBs was only in the range of 0.0726 to 0.0952 W/(m K) for a temperature range of 24.0 °C to 80.0 °C. However, adding DPSF to the BTBs forms hybrid composite materials with enhanced thermal conductivity coefficient for samples # 5, 6, and 7 from 0.056 to 0.070 W/(m K), 0.062 to 0.074 W/(m K), and 0.054 to 0.066 W/(m K) compared to the bound composite BTBC # 2, for the same temperature range, respectively. The noise reduction coefficient is greater than 0.35 for composite samples # 2, 5, 6, and 7 using the average value of a one-third octave. The composite sample boards are thermally stable up to 291.0 °C. Furthermore, the tested composite specimens’ flexure modulus, flexural stress, and flexural strain have an average value of 0.21–10.5 MPa, 0.12–0.37 MPa, and 0.01–0.40, respectively. Moreover, the newly developed samples proved to have a low percentage of moisture content below 2.2%. These promising conclusions encourage using these composite materials for thermal insulation and sound absorption in buildings since they are biodegradable, eco-friendly, and natural. Moreover, using such newly developed composites will reduce the negative environmental impacts. In addition to that, these composites offer several advantages, such as low cost, availability of renewable natural resources, and biodegradability over synthetic and petrochemical materials.

## Figures and Tables

**Table 1:** Complete dimensions, mass, percentage, volume, and density of the prepared samples.

Material Specifications	Sample Number								
	BTBP (# 1)	BTBC (# 2)	DPSFP (# 3)	DPSFC (# 4)	COMP (# 5)	COMP (# 6)	COMP (# 7)	COMP (# 8)	COMP (# 9)
BTB %	100	65.4	0.0	0.0	64	39.0	19.0	13.5	34.0
DPSF %	0.0	0.0	100	81.3	21	39.0	57.0	40.5	34.0
Ratio of polymerized (PVA) resin to total mass %	0.0	34.6	0.0	18.7	15.0	22.0	24.0	46.0	32.0
Thickness, δ (mm)	41.0	18.0	46.0	46.0	25.0	32.0	42.0	25.0	29.0
The sample’s volume (cm^3^)	3330	1620	4132	4140	2250	2880	3780	2250	2610
BTB’s mass (g)	439	500	0.0	0.0	375	250	125	100	250
DPSF’ mass (g)	0.0	0.0	500	500	125	250	375	300	250
Resin’s mass (g)	0.0	265	0.0	115	86	148	158	341	234
Figure #	2(a)	5(a)	2(b)	5(b)	5(c)	5(d)	5(e)	5(f)	5(g)
Apparent density of dried samples (kg/m^3^)	132	472	121	149	260	225	174	329	281
Total dried mass (g)	439	765	500	615	586	648	658	741	734

**Table 2:** Bending specimens’ specifications.

Specimens’ Number	Thickness (δ) (mm)	Width (*b*) (mm)	Span (*L*) (mm)	Slope (*S*) (N/mm)
2	18.0	47.0	150.0	3.2
4	46.0	51.0	150.0	1.4
5	25.0	55.0	150.0	7.9
6	32.0	53.0	150.0	0.45
7	42.0	51.0	150.0	1.7
8	25.0	53.0	160.0	49.0
9	29.0	51.0	160.0	5.2

**Table 3:** Flexure Modulus, Flexural Stress, and Flexural Strain of the tested specimens.

Fabricated Specimen No.	Slope (S) (N/mm)	Thickness (δ) (mm)	Density, (kg/m^3^)	Polymerized (PVA) Resin to the Total Mass %	Flexure Modulus (MPa), *E_f_*	Flexural Stress (MPa), *σ_f_*	Flexural Strain at Flexural Strength, *ε_f_*
2	3.2	18.0	472.0	34.6	10.5 ± 1.4	0.16 ± 0.01	0.01 ± 0.0005
4	1.4	46.0	149.0	18.7	0.24 ± 0.03	0.12 ± 0.01	0.4 ± 0.018
5	7.9	25.0	260.0	15.0	7.6 ± 0.98	0.37 ± 0.03	0.05 ± 0.002
6	0.45	32.0	225.0	22.0	0.21 ± 0.03	0.06 ± 0.005	0.25 ± 0.011
7	1.7	42.0	174.0	24.0	0.39 ± 0.05	0.10 ± 0.008	0.33 ± 0.015
8	49.0	25.0	329.0	46.0	68.9 ± 8.87	1.52 ± 0.13	0.02 ± 0.0009
9	5.2	29.0	281.0	32.0	4.3 ± 0.55	0.16 ± 0.014	0.02 ± 0.0008

**Table 4:** Linear fitting equations for the thermal conductivity coefficient profiles given in Figure 13.

Equation (3)	k = A + B × t				
Sample #	Symbol	A	B	R^2^, %	Density, kg/m^3^
1 (BTBP)	♦	0.049	0.0002	92.6	132.0
2 (BTBC)	●	0.064	0.0004	98.7	472.0
3 (DPSFP)	×	0.046	0.0002	97.2	121.0
4 (DPSFC)	ο	0.054	0.0003	97.4	149.0
5 (COMP)	▲	0.052	0.0002	99.8	260.0
6 (COMP)	■	0.059	0.0002	93.0	225.0
7 (COMP)	Δ	0.049	0.0002	94.0	174.0
8 (COMP)	□	0.057	0.0003	99.7	329.0
9 (COMP)	+	0.057	0.0003	98.1	281.0

**Table 5:** Comparison between the study’s developed boards and the conventional synthetic materials.

Materials	Density (kg/m^3^)	Thermal Conductivity (W/mK)	References
BTBP (# 1)	132	0.05405–0.0666	This study
BTBC (# 2)	472	0.072636–0.095211	This study
DPSFP (# 3)	121	0.048461–0.059728	This study
DPSFC (# 4)	149	0.060877–0.078064	This study
COMP (# 5)	260	0.05597–0.0703	This study
COMP (# 6)	225	0.0617–0.0741	This study
COMP (# 7)	174	0.0543–0.0655	This study
COMP (# 8)	329	0.062125–0.079914	This study
COMP (# 9)	281	0.063792–0.081400	This study
Recycled polyethylene terephthalate (PET)	30	0.0355	[36]
Recycled (PET) (commercialized)	15–60	0.034–0.039	[35]
Recycled glass fibers (commercialized)	100–165	0.038–0.050	[35]
Polyurethane foam	30–80	0.02–0.027	
Rock wool	40–200	0.033–0.040	[35]
Expanded polystyrene (XPS)	15–35	0.031–0.038	[35]
Extruded polystyrene (EPS)	32–40	0.032–0.037	[35]
Kenaf	30–180	0.034–0.043	[35]
Sheep wool	10–25	0.038–0.054	[35]

**Table 6:** Noise reduction coefficient for samples # 2, 4, 5, 6, and 7.

Sample Number	Density, kg/m^3^	Frequency (Hz)				NRC
		250	500	1000	2000	
		Sound Absorption Coefficients (SAC)				
2	472	0.0397	0.1162	0.6149	0.7098	0.35
4	149	0.0736	0.1459	0.3029	0.1683	0.15
5	260	0.054	0.0933	0.391	0.9402	0.35
6	225	0.1097	0.2862	0.8149	0.5376	0.45
7	174	0.0818	0.1736	0.5362	0.7534	0.40

**Table 7:** Comparison of SAC and NRC with some similar materials in the literature.

Materials	Density (kg/m^3^)	Thickness of the Board or Fiber (m)	Sound Absorption Coefficient, SAC				NRC	References
			Frequency, Hz					
			250	500	1000	2000		
BTBC (# 2)	472	0.018	0.0397	0.1162	0.6149	0.7098	0.35	This study
DPSFC (# 4)	149	0.046	0.0736	0.1459	0.3029	0.1683	0.15	This study
COMP (# 5)	260	0.025	0.054	0.0933	0.391	0.9402	0.35	This study
COMP (# 6)	225	0.032	0.1097	0.2862	0.8149	0.5376	0.45	This study
COMP (# 7)	174	0.042	0.0818	0.1736	0.5362	0.7534	0.40	This study
Polyurethane foam	95	------------	0.02	0.01	0.11	0.16	0.08	[37]
Kenaf (light)	50	0.06	0.19	0.33	0.68	0.9	0.55	[24]
Wood (fibers)	100	0.04	0.40	0.50	0.65	0.91	0.60	[24]
Coconut	60	0.04/0.06	0.2	0.34	0.67	0.79	0.50	[24]
Cork	100	0.03	0.02	0.10	0.30	0.86	0.30	[24]
Cane (only wooden)	400	0.04	0.06	0.12	0.47	0.43	0.25	[24]
Fleece (100% polyester) fiber	60	0.0035	0.08	0.12	0.19	0.21	0.15	[42]
Queenscord fiber	160	0.0019	0.05	0.14	0.34	0.30	0.20	[42]
Mesh fiber	100	0.0033	0.18	0.02	0.05	0.07	0.10	[42]
Suede fiber	300	0.0006	0.09	0.13	0.24	0.28	0.20	[42]
Wood fiberboard	480	0.018	0.11	0.14	0.21	0.34	0.20	[43]
Palm oil leaves	152	0.010	---	0.05	0.08	0.19	0.10	[44]
Lemongrass	201	0.010	---	0.06	0.15	0.45	0.20	[44]

**Table 8:** Degradation of tea bag components and hybrid composite sample # 7.

**Tea Bag,** 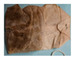							
**Thermally Stable Zone**		**T_50%_ Degradation**		**End of Major Degradation**		**Char Formation**	
Mass % 90	Temp. (°C) 284.5	Mass % 50	Temp. (°C) 349.0	Mass % 24.3	Temp. (°C) 379.5	Mass % 16	Temp. (°C) 548.0
**String,** 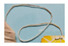							
Mass % 90	Temp. (°C) 287.0	Mass % 50	Temp. (°C) 351.0	Mass % 24	Temp. (°C) 378.0	Mass % 15	Temp. (°C) 547.0
**Label,** 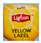							
Mass % 90	Temp. (°C) 300.7	Mass % 50	Temp. (°C) 473.0	Mass % 56	Temp. (°C) 368.6	Mass % -------	Temp. (°C) -------
**Composite Sample # 7,** 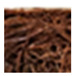							
Mass % 90	Temp. (°C) 291.0	Mass % 50	Temp. (°C) 413.5	Mass % 34	Temp. (°C) 487.0	Mass % 20.4	Temp. (°C) 800.0

## Data Availability

The original contributions presented in the study are included in the article, further inquiries can be directed to the corresponding author.

## Nomenclatures

A	Constant
B	Constant
BTBP	Black tea bag polymer (sample # 1)
BTBC	Black tea bag composite (sample # 2)
COMP	Composite sample of BTB, DPSF, and the binder
DPSFP	Date palm surface fiber polymer (sample # 3)
DPSFP	Date palm surface fiber composite (sample # 4)
DTGA	Differential thermogravimetric analysis
k	Thermal conductivity coefficient
NRC	Noise reduction coefficient
SAC	Sound absorption coefficient
S	Slope
t	Temperature (°C)
TGA	Thermogravimetric analysis
